# Infection and Coinfection of Human Rhinovirus C in Stem Cell Transplant Recipients

**DOI:** 10.1155/2013/236081

**Published:** 2013-02-27

**Authors:** Filippo Canducci, Maurizia Debiaggi, Elisa Rita Ceresola, Michela Sampaolo, Emilio Paolo Alessandrino, Roberto Brerra, Aurora Piazza, Massimo Clementi

**Affiliations:** ^1^Università degli studi dell'Insubria, Via Ravasi 2, 21100 Varese, Italy; ^2^University of Pavia, Corso Strada Nuova 65, 27100 Pavia, Italy; ^3^Vita-Salute San Raffaele University, Via Olgettina 58, 20132 Milan, Italy; ^4^Fondazione IRCCS San Matteo Hospital, Viale Camillo Golgi 19, 21100 Pavia, Italy

## Abstract

In 54 adult stem cell transplant recipients, the presence and persistence of human rhinoviruses (including the novel lineage C) were evaluated by molecular detection and phylogenetic analysis, independently from respiratory symptoms. In the same group of patients, the presence of other coinfecting respiratory pathogens, including the novel enterovirus 109, was also evaluated.

## 1. Introduction

Human rhinoviruses (HRVs) are common agents of human infections [1]. Due to their similar genomic organization, they are classified within the same family *Picornaviridae* together with the human enteroviruses (HEV) [2, 3]. However, the common genomic features reflect modestly the characteristics of these viruses in terms of tropism and pathogenic potential. HRVs generally cause mild infections, replicating in the upper respiratory tract, but, in infants and immunocompromised patients, HRVs can reach the lower respiratory tract causing serious illnesses and act as pathogens or copathogens in bronchitis, bronchiolitis, and pneumonia [4, 5]. Recently a new HRV lineage designated HRV-C has been identified using molecular methods and associated with severe clinical presentations in infants and immunocompromised adults [6]. The majority of HEVs instead replicate in the gastrointestinal tract, eventually spreading to other organs such as the heart or the central nervous system giving rise to serious diseases. Only a few HEV genotypes can occasionally be identified in the respiratory secretions of patients with lower respiratory tract infections [5, 7]. Moreover, a novel HEV (HEV 104) was identified in Switzerland and later in other countries often in association with respiratory signs [7, 8]. Finally, a novel HEV (HEV 109) was molecularly identified in the nose and throat swab samples collected between June 2007 and June 2008 from children enrolled in a cohort study of influenza-like illness in Nicaragua, in some cases also associated with enteric symptoms [9].

In the present study, the prevalence and persistence and the pathogenic potential of HRVs were retrospectively evaluated by molecular analysis and phylogenetic reconstruction in sequential nasopharyngeal aspirates obtained from immunosuppressed hematopoietic stem cell transplantation adult recipients. In the same cohort the presence of other coinfecting respiratory pathogens, including the novel HEV 109, was also evaluated.

## 2. Methods

A total of 175 archived respiratory samples (nasopharyngeal aspirates) were obtained from 50 allogeneic and 4 autologous haematopoietic stem cell transplant recipient patients recruited at the Division of Hematology, IRCCS Policlinico San Matteo (Pavia, Italy) regardless of respiratory symptoms, as described [10, 11]. Respiratory samples were consecutively obtained after informed consent from October 1st, 2004 to April 2007. From one to nine samples from each patient were collected every 30 days up to 180 days after transplantation. At each time point all the clinical data were collected. RNA was purified from all samples using the Qiagen RNA mini kit (Qiagen, Germany) following the manufacturer protocol. To investigate HRV infections, all samples, were subjected to first round amplification after sample extraction using Primers P1-1 (CAA GCA CTT CTG TYW CCC C) and P3-1 (ACG GAC ACC CAA AGT AG); and semi-nested amplification was performed using forward primer P1-1 and three reversed primers P2-1 (CAA GCA CTT CTG TYW CCC C), P2-2 (TTA GCC ACA TTC AGG AGC C) and P2-3 (TTA GCC GCA TTC AGG GG) as described previously [12]. Since HEV 109 was initially cross-amplified also with the previous amplification protocol (designed to amplify the widest number of Picornaviruses), we decided to further evaluate HEV 109 prevalence by using specific primers targeting the NTR region of HEV 109 (EV109 VP1 123F, 5′-GGA GAC TGG AGC AAC TAG TAA AG-3′; EV109 VP1 363R, 5′-GGT GAA CAT TTC CAA TTT CCT ACG-3′).

All the amplification products were sequenced bidirectionally to confirm amplification specificity and virus typing by phylogenetic analysis. Molecular identification and typing of enterovirus and rhinovirus positive samples were performed with MEGA 3.1 software after ClustalW alignment and manual sequence editing with BioEdit. Phylogenetic relationships were estimated using MEGA V3.1 (neighbor-joining method by using Tajima-Nei model as estimated by using Modeltest; the *α* value used in MEGA was previously estimated directly from the data by using PAUP).

Since multiple infections are frequently detected in respiratory samples of patients with respiratory symptoms and to better clarify the pathogenetic role of picornaviruses in coinfections, all specimens positive for HRVs or HEV 109, were also assayed for the presence of other thirteen respiratory viruses, including parainfluenza viruses (PIV 1–3), influenza A and B viruses, human metapneumovirus (hMPV), human respiratory syncytial virus (hRSV), and adenoviruses, using a multiplex PCR strategy (Seeplex RV12 ACE Detection, Seegene, Rockville). Moreover, rhinovirus positive samples were also tested for human bocaviruses (hBoV), human coronaviruses (hCoV) and of the recently identified WU and KI polyomaviruses, using protocols described previously [13, 14].

## 3. Results and Discussion

Thirty seven out of the 175 samples (from 23 transplant patients) tested positive for HRV infection.

**Figure 1:**
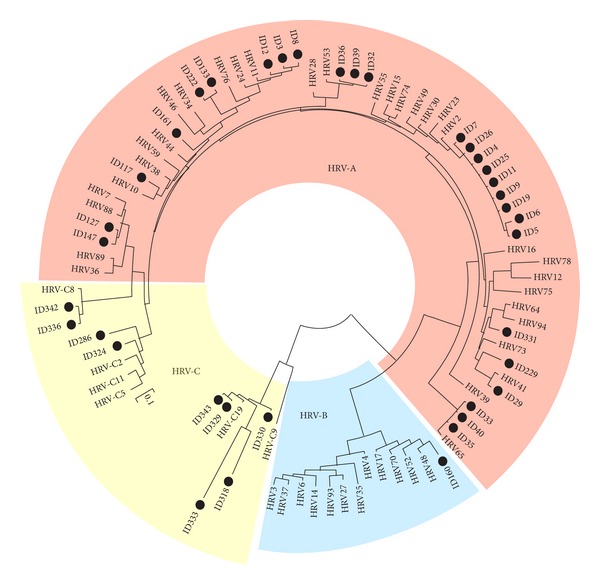
Genotyping of human rhinoviruses amplified from transplant patients. Genetree of human rhinovirus VP1 sequences. Reference sequences were obtained from the Picornavirus Study Group database (http://www.picornastudygroup.com/). Samples amplified from patients are indicated by a filled black circle.

Eight of the 23 HRV positive patients were also coinfected by one or more respiratory virus (Table 1); thirteen of the 23 patients were asymptomatic. Subtyping of HRV samples allowed to identify 15 HRV-A strains, 1 HRV-B strain, and 7 HRV belonging to the novel C lineage (Table 1 and Figure 1). In 2 out of the 7 HRV-C samples coinfections with hMPV or hCoV were identified and both were symptomatic. All of the other monoinfected HRV-C positive samples were asymptomatic. Finally, 8 out of the 15 HRV-A strains and the HRV-B strains resulted to be associated with upper respiratory symptoms such as rhinorrhea, pharingodinia or tussis, being 4 of them coinfected with other pathogens.

The novel HEV 109 could be identified in a symptomatic immunocompromised adult from a respiratory sample collected at the beginning of 2006. The tedious pharngodynia suffered by this patient in the absence of other coinfecting respiratory viruses suggests a direct pathogenetic role of this virus as speculated in initial observations [9]. Overall, HRV infections were common and frequently asymptomatic in this group of patients, including the majority of rhinoviruses infections due to HRV-C strains. In two patients with rhinorrhea, the HRV-C strains were detected together with other coinfecting viral pathogens. These results do not confirm the previously observed high pathogenic potential of this lineage, at least in immunocompromised hosts [6].

As previously documented for hMPV infections in immunocompromised patients [10, 11], not only sequential infections by different HRV subtypes were identified (patients 10 and 16), but long (up to 45 days) viral shedding was observed in four out of 23 patients (patients 1, 3, 18, and 22) not constantly associated with symptoms. Nevertheless as recently suggested, symptomatic or asymptomatic viral infections can trigger acute rejection and obliterative bronchiolitis in lung transplant recipients and warrant a continuous monitoring in immunocompromised patients, with methods able to identify an always wider panel of potential viral pathogens [15].

## Figures and Tables

**Table 1:** Picornavirus positive samples. Samples' ID (as shown in the phylogenetic tree) and time of collection are shown. Result of phylogenetic subtyping, coinfections, and respiratory symptoms are also reported.

Patient	Sample ID	Days from transplantation	Picornavirus subtype	Coinfections	Respiratory symptoms
1	ID4	15	HRV-A2	hMPV, hCoV	No
	ID5	25	HRV-A2	hMPV	No
	ID9	35	HRV-A2		No
2	ID333	30	HRV-C19	hMPV	Rhinorrhea
3	ID6	−5	HRV-A2	hMPV, hCoV	No
	ID7	15	HRV-A2		No
	ID11	35	HRV-A2	hMPV	No
4	ID26	15	HRV-A2		No
5	ID229	60	HRV-A73/41		Pharingodinia
6	ID33	−30	HRV-A65		Rhinorrhea
7	ID318	0	HRV-C19		No
8	ID331	60	HRV-A64/94		No
9	ID336	15	HRV-C8		No
10	ID19	−5	HRV-A2	hMPV	No
	ID40	30	HRV-A65		Rhinorrhea
	ID29	60	HRV-A41		Rhinorrhea
11	ID343	30	HRV-C19		No
12	ID35	−5	HRV-A65	hMPV	Rhinorrhea
	ID25	0	HRV-A2	hMPV, hCoV	Rhinorrhea
13	ID342	90	HRV-C8	hCoV	Rhinorrhea
14	ID39	15	HRV-A53		Tussis
	ID32	30	HRV-A53		No
15	ID329	15	HRV-C19		No
16	ID133	15	HRV-A65		No
	ID160	60	HRV-B		Rhinorrhea
17	ID286	0	HRV-C2		No
18	ID127	15	HRV-A88		No
	ID147	60	HRV-A88		Pharyngodynia
19	ID222	120	HRV-A76		No
20	ID161	90	HRV-A44		No
21	ID117	90	HRV-A10	PIV3	Rhinorrhea
22	ID3	−5	HRV-A11	hMPV	No
	ID8	15	HRV-A11	hMPV	No
	ID12	30	HRV-A11	hMPV	No
23	ID36	0	HRV-A53		No
24	ID175	180	HEV-109		Pharingodinia

hMPV: human metapneumovirus; hCoV: human coronaviruses; PIV3: parainfluenza virus type 3.
